# North American Lauraceae: Terpenoid Emissions, Relative Attraction and Boring Preferences of Redbay Ambrosia Beetle, *Xyleborus glabratus* (Coleoptera: Curculionidae: Scolytinae)

**DOI:** 10.1371/journal.pone.0102086

**Published:** 2014-07-09

**Authors:** Paul E. Kendra, Wayne S. Montgomery, Jerome Niogret, Grechen E. Pruett, Albert E. Mayfield, Martin MacKenzie, Mark A. Deyrup, Gary R. Bauchan, Randy C. Ploetz, Nancy D. Epsky

**Affiliations:** 1 United States Department of Agriculture, Agricultural Research Service, Subtropical Horticulture Research Station, Miami, Florida, United States of America; 2 Archbold Biological Station, Lake Placid, Florida, United States of America; 3 United States Department of Agriculture, Forest Service, Southern Research Station, Asheville, North Carolina, United States of America; 4 United States Department of Agriculture, Forest Service, Forest Health Protection, Stanislaus National Forest, Sonora, California, United States of America; 5 United States Department of Agriculture, Agricultural Research Service, Beltsville Area Research Center, Electron and Confocal Microscopy Unit, Beltsville, Maryland, United States of America; 6 University of Florida, Tropical Research and Education Center, Homestead, Florida, United States of America; Swedish University of Agricultural Sciences, Sweden

## Abstract

The invasive redbay ambrosia beetle, *Xyleborus glabratus*, is the primary vector of *Raffaelea lauricola*, a symbiotic fungus and the etiologic agent of laurel wilt. This lethal disease has caused severe mortality of redbay (*Persea borbonia*) and swampbay (*P. palustris*) trees in the southeastern USA, threatens avocado (*P. americana*) production in Florida, and has potential to impact additional New World species. To date, all North American hosts of *X. glabratus* and suscepts of laurel wilt are members of the family Lauraceae. This comparative study combined field tests and laboratory bioassays to evaluate attraction and boring preferences of female *X. glabratus* using freshly-cut bolts from nine species of Lauraceae: avocado (one cultivar of each botanical race), redbay, swampbay, silkbay (*Persea humilis*), California bay laurel (*Umbellularia californica*), sassafras (*Sassafras albidum*), northern spicebush (*Lindera benzoin*), camphor tree (*Cinnamomum camphora*), and lancewood (*Nectandra coriacea*). In addition, volatile collections and gas chromatography-mass spectroscopy (GC-MS) were conducted to quantify terpenoid emissions from test bolts, and electroantennography (EAG) was performed to measure olfactory responses of *X. glabratus* to terpenoids identified by GC-MS. Significant differences were observed among treatments in both field and laboratory tests. Silkbay and camphor tree attracted the highest numbers of the beetle in the field, and lancewood and spicebush the lowest, whereas boring activity was greatest on silkbay, bay laurel, swampbay, and redbay, and lowest on lancewood, spicebush, and camphor tree. The Guatemalan cultivar of avocado was more attractive than those of the other races, but boring response among the three was equivalent. The results suggest that camphor tree may contain a chemical deterrent to boring, and that different cues are associated with host location and host acceptance. Emissions of α-cubebene, α-copaene, α-humulene, and calamenene were positively correlated with attraction, and EAG analyses confirmed chemoreception of terpenoids by antennal receptors of *X. glabratus*.

## Introduction

Laurel wilt is a destructive vascular disease of American trees in the family Lauraceae, particularly members of the genus *Persea*. Over the last decade, large populations of native redbay and swampbay [*P. borbonia* (L.) Spreng. and *P. palustris* (Raf.) Sarg., respectively] have been decimated throughout the southeastern United States [1]–[2], and currently avocado (*P. americana* Mill.) is threatened in south Florida [3]. The disease emerged subsequent to establishment of the redbay ambrosia beetle, *Xyleborus glabratus* Eichhoff (Coleoptera: Curculionidae: Scolytinae), an invasive wood borer native to Southeast Asia [4]. Female beetles store several fungal symbionts in cuticular pouches (mycangia) at the base of the mandibles, one of which, *Raffaelea lauricola* T. C. Harr., Fraedrich & Aghayeva (Ophiostamatales: Ophiostomataceae), causes laurel wilt [5]–[6]. The presence of *R. lauricola* in susceptible hosts elicits a cascade of events, including secretion of resins and formation of extensive parenchymal tyloses that wall off conductive xylem vessels [7]–[8]. This defensive response results in diminished water transport, which initially impedes spread of the mycopathogen, but ultimately leads to systemic wilt and host tree mortality. A recent study documented lateral transfer of *R. lauricola* to other species of ambrosia beetle (secondary colonizers that breed sympatrically with *X. glabratus*), and transmission of the pathogen to hosts under laboratory conditions [9], but it is not yet known if these additional species contribute to the spread of laurel wilt in natural ecosystems.

The first redbay ambrosia beetle detected in North America was trapped in May 2002 in a maritime port near Savannah, Georgia [10]. Since that time, its geographic range has expanded at a rate exceeding model predictions [11]. Southward spread through the Florida peninsula was particularly rapid, due to mild temperatures, ample host availability, and human transport of infested material (e.g. firewood [12]). In March 2010, five years before the predicted date [11], *X. glabratus* had reached southernmost Florida (Miami-Dade County); this was followed by confirmation of laurel wilt in the county by 2011 [13] and in commercial avocado groves by 2012 [3]. As of February 2014, the vector-pathogen complex had been confirmed in portions of six southeastern states: North Carolina, South Carolina, Georgia, Florida, Alabama, and Mississippi [14], but further expansion is likely (see [15]–[16] for recent reviews of the epidemic). Avocado production provides an estimated $23.5 million in sales annually for the state of Florida [17]. With continued spread, laurel wilt could pose a serious economic threat to the avocado industries in California ($468 million in annual sales [17]) and Mexico ($1.2 billion in export revenue alone [18]). Moreover, there is potential for severe ecological impact on forest ecosystems in Mexico, Central and South America, areas rich in species of *Persea* and other genera within the Lauraceae [16], [19].

Development of effective semiochemical-based detection and control programs for *X. glabratus* will require an understanding of its unique chemical ecology. Like other ambrosia beetles within the tribe Xyleborini, *X. glabratus* is known to have extensive inbreeding, haplo-diploid sex determination, and a sex ratio highly skewed toward females [10]. Species-specific pheromones are not used, as females typically mate with flightless, sibling males prior to emergence from natal trees. However, females of *X. glabratus* are behaviorally atypical for this taxonomic group. While most xyleborines are broad generalists that target stressed or dying trees (saprotrophic symbiosis) [20], *X. glabratus* functions ecologically as a primary colonizer, capable of attacking live, apparently-healthy hosts [1]. Consequently, it is not attracted to ethanol [2], [21], a signature volatile of tree decay, which serves as the standard lure for detection of most ambrosia beetles [22]. In addition, *X. glabratus* is not a host generalist in the United States, but appears to be restricted to the Lauraceae. To date, at least 12 U.S. species – all in the Lauraceae – have been reported as either hosts of *X. glabratus* and/or suscepts of laurel wilt [1]–[2], [23]–[27]. It was thought that this host specificity represented a major behavioral shift that accompanied establishment of the founder population in North America [15], but a recent inspection of host records from the Chinese National Insect Collection suggests that *X. glabratus* shows a preference for Lauraceae in Asia as well [28].

It is not a strict specialist, however, since reported hosts do include representatives from other families, including Dipterocarpaceae, Fabaceae, Fagaceae, Theaceae, and Pinaceae [10], [28]. In Florida, it was discovered that *X. glabratus* is highly attracted to, and will initiate boring into, freshly-cut branches (bolts) of lychee, *Litchi chinensis* Sonn. (Sapindaceae) [29]. Subsequent evaluations indicated that *L. chinensis*, although attractive to *X. glabratus* due to chemical similarities with the Lauraceae, is not a suitable reproductive host. This is apparently due to the inability of lychee wood to support growth of *R. lauricola*, the presumed primary nutritional symbiont [30]. This latter work, in combination with other field studies of *X. glabratus*, provides evidence that dispersing females locate potential host trees based on their volatile emissions. Our current hypothesis is that *X. glabratus* detects (via antennal olfactory receptors) a mixture of terpenoid compounds in which the sesquiterpene α-copaene functions as a key long-range attractant [29]–[35]. These terpenoids – tantamount to a signature bouquet of the Lauraceae – have been found to be concentrated in several plant-derived essential oils, including phoebe, manuka, and cubeb oils, which have been utilized as field lures for detection of *X. glabratus* [21], [29], [31], [34]–[35].

The present study was initiated to investigate in-flight attraction and boring preferences of female *X. glabratus* for the dominant species of Lauraceae in the U.S., and to evaluate the relationship between behavioral response and phytochemical emissions from wood substrates. Of the ∼50 described genera within the Lauraceae, only nine occur in North America: *Cassytha*, *Cinnamomum*, *Licaria*, *Lindera*, *Litsea*, *Nectandra*, *Persea*, *Sassafras*, and *Umbellularia* [36]. We compared responses of *X. glabratus* to cut bolts from nine tree species, representative of six of these genera. *Cassytha* was excluded from the study because it is a non-woody, parasitic vine [36] not susceptible to attack by a wood boring beetle. *Litsea* and *Licaria* were also omitted since they are rare plant species of conservation concern in the U.S.; however, both genera have been evaluated previously for susceptibility to laurel wilt [23], [27]. Specific components of our study included (i) field tests to determine relative attraction among the nine species of Lauraceae, (ii) laboratory bioassays to assess female boring behavior as an indicator of host recognition and acceptance, (iii) volatile collections followed by gas chromatography-mass spectroscopy (GC-MS) to quantify terpenoid emissions from the bolt treatments, and (iv) electroantennography (EAG) to measure the beetle's peripheral olfactory response to the major constituents identified by GC-MS. Chemicals of primary interest were volatile compounds previously reported as potential attractants for *X. glabratus*, including α-copaene and several other sesquiterpene hydrocarbons [21], [29]–[35], [37]–[38], as well as the monoterpene ether eucalyptol (1,8-cineole) [37], [39].

## Materials and Methods

### Ethics Statement

Field studies were conducted at the Lake Wales Ridge Environmental Management Area under special use permit #SUO-33630 issued by the Florida Fish and Wildlife Conservation Commission. Test bolts from Matheson Hammock were collected under research permit #148 from the Miami-Dade County Parks and Recreation Department. Collection of bolts from other sites did not require specific permits; however, verbal permission was obtained from, and collection activities were coordinated with appropriate curators, land stewards, and rangers (listed in the acknowledgments). Field studies did not involve any protected or endangered species.

### Test Substrates

Plant material for field tests, bioassays, and chemical analyses consisted of freshly-cut bolts (15–20 cm long and 5–8 cm in diameter) obtained from multiple locations: avocado cultivars ‘Catalina’ (West Indian race; MIA# 17248, PI# 281923, WA2-18-34), ‘Duke’ (Mexican race; MIA# 17468, PI# 277487, WA4-28-51), and ‘Taylor’ (Guatemalan race; MIA# 18262, PI# 26710, WB3-13-02) from the National Germplasm Repository at the USDA-ARS Subtropical Horticulture Research Station (SHRS; Miami, FL); redbay, swampbay, silkbay (*Persea humilis* Nash), and live oak (*Quercus virginiana* L.) from Archbold Biological Station (ABS; Lake Placid, FL); lancewood [*Nectandra coriacea* (Sw.) Griseb.] from Matheson Hammock (Coral Gables, FL); camphor tree [*Cinnamomum camphora* (L.) J. Presl] from Bok Tower Gardens (Lake Wales, FL); sassafras [*Sassafras albidum* (Nutt.) Nees] and northern spicebush [*Lindera benzoin* (L.) Blume] from Bent Creek Experimental Forest (Asheville, NC); and California bay laurel [*Umbellularia californica* (Hook. & Arn.) Nutt.] from Stanislaus National Forest (Tuolumne County, CA). Since California bay laurel is a foliar host of the oomycete plant pathogen *Phytophthora ramorum* Werres, DeCock & Man in't Veld (Pythiales: Pythiaceae), the causal agent of sudden oak death [40], bolts from bay laurel were collected outside the quarantine zone for *P. ramorum*; in addition, bolts from this site were assayed and found negative for the pathogen prior to shipment to Florida, as described previously for parallel tests conducted concurrently in South Carolina [25]. At time of collection, the ends of each bolt were wrapped in Parafilm M (Pachiney Plastic Packaging, Inc., Chicago, IL, USA) to minimize desiccation and loss of volatile phytochemicals. Samples from California and North Carolina were packed in insulated coolers and shipped overnight to Florida. At test deployment, a thin section (∼0.5 cm) was cut from the ends of each bolt using a battery operated reciprocating saw (Craftsman; Sears, Roebuck and Co., Chicago, IL, USA). All tests were initiated within 3 days of bolt collection.

### Field Tests

Trapping experiments were conducted in south-central Florida (Lake Placid, Highlands County) at two tracts within the Lake Wales Ridge Environmental Management Area. Both sites had numerous swampbay trees exhibiting advanced stages of laurel wilt, but there were also silkbay trees (mostly asymptomatic) in adjacent dry scrub habitats. Test 1 was conducted at the Royce Ranch Unit (N 27°38′410″, W 81°34′307″) from 24 September to 12 November 2010 (7-wk test), and evaluated attraction to five species of Lauraceae: redbay, swampbay, silkbay, avocado, and lancewood. In addition, the test included two controls, consisting of an unbaited trap (to assess random background captures) and a trap baited with live oak (a non-host bolt treatment, to assess potential captures resulting from visual cues [41]). Test 2 was conducted at the Highlands Park Estates (N 27°21′032″, W 81°19′837″) from 4 August to 15 September 2011 (6-wk test), and compared captures of *X. glabratus* with bolts of camphor tree, sassafras, northern spicebush, California bay laurel, silkbay (an internal control for comparison with results from test 1), and an unbaited trap.

Trap design consisted of two bolts wired together (side-by-side) and hung vertically, to which were attached two white sticky panels (23×28 cm, Sentry wing trap bottoms; Great Lakes IPM, Vestaburg, MI, USA) stapled back-to-back to the bottom of the bolts. The paired sticky panels were secured further with several binder clips around the edges. The unbaited control traps consisted of two sticky panels stapled together. Field tests followed a randomized complete block design, with ten replicate blocks in test 1, and five replicate blocks in test 2. Replicate bolts for each host species were obtained from different trees. In field test 1, the ten replicate bolts of avocado consisted of four bolts of the Mexican cultivar and three bolts each of the West Indian and Mexican cultivars. Each block consisted of a row of traps hung from wire hooks ∼1.5 m above ground [42] in non-host trees, with a minimum of 10 m spacing between adjacent traps in a row, and 30 m spacing between rows. For both tests, traps were checked weekly. At each sampling date, the sticky panels were collected, a thin layer was sawed from the bottom of each bolt to “renew” release of wood volatiles, new sticky panels were attached, and the trap positions were rotated sequentially within each row (block). The latter step ensured that each treatment was rotated through each of the field positions within a block, thereby minimizing positional effects on beetle capture.

All sample collections were sorted under a dissecting microscope in the laboratory at SHRS. Species of Scolytinae were removed from the sticky panels, soaked in histological clearing agent (Histo-clear II; National Diagnostics, Atlanta, GA, USA) to remove adhesive, and then stored in 70% ethanol. Beetles were identified according to Rabaglia et al. [10] and voucher specimens were deposited at SHRS and ABS.

### Test Insects

Insects used in laboratory bioassays and electrophysiology studies were host-seeking female *X. glabratus* collected in the field (at ABS and several sites along the Lake Wales Ridge Ecosystem) using a published baiting method [43]. The procedure used freshly-cut *Persea* wood and several manuka oil lures (Synergy Semiochemicals Corp., Burnaby, BC, Canada) as bait placed in the center of a white cotton sheet. At 15–20 min intervals, fresh wood was added to the pile and the lures were fanned. This generated a pulsed plume of attractive volatiles that effectively ‘lured in’ host-seeking females, which have peak flight from 17:30–19:30 h (EDST) in south Florida [33]. As beetles landed, they were collected by hand with a soft brush and placed in plastic boxes containing moist tissue paper. Insects were then held overnight in the storage boxes until used in laboratory experiments early the next morning.

To visualize the fine morphological features of the *X. glabratus* antenna (the primary olfactory organ used by insects for chemoreception and transduction of environmental odors), several females were examined by low-temperature scanning electron microscopy (LT-SEM), using methods recently reported [44].

### Laboratory Bioassays

Behavioral bioassays consisted of no-choice tests designed to document host recognition and boring behaviors, following published protocols [29]–[30]. Assays were conducted at ABS under controlled laboratory conditions (25°C, 16:8 h L:D). Test arenas consisted of plastic buckets (4.4 liter) covered with cheese cloth mesh, secured with rubber bands, and held in screened insect cages (BioQuip, Rancho Dominguez, CA, USA). Into each bucket, lined with a filter paper disk (15 cm diameter; Whatman Intl. Ltd., Maidstone, England), were placed 10–15 beetles and a single bolt of wood. Bolt treatments were identical to those evaluated in the field tests. A beetle was scored positive for boring when it was perpendicular to the wood substrate and at least half its body length (∼1 mm) was inserted into the entrance hole. (Previous observations indicated that females sometimes ‘sampled’ the substrate, making shallow bore holes, but then aborted the attempt; however, once they inserted half their body length, they typically continued to bore through the bark and cambium layers into the sapwood.) To document behavioral response over time, the number of beetles that were boring and their location on the bolt were recorded at 1, 2, 4, 8, 12, and 24 h. Tests were replicated a minimum of five times for each substrate, and each replicate was run using a separate arena, a new bolt of wood, and a new cohort of beetles.

### Chemical Collection and Analysis

Samples for chemical analysis were prepared by manually rasping the outer layers of bark and underlying cambial tissue from the bolt treatments, using methods reported previously [32], [45]. Volatile chemicals were collected from freshly-rasped shavings (6 g samples, 3–10 replicates per species) by using Super Q traps (Analytical Research Systems, Gainesville, FL, USA) according to published methods [46]–[47]. Samples were spread in a cylindrical glass chamber (4.5 cm diameter ×25 cm length), purified air was introduced into the chamber (1 L/min), and headspace volatiles were collected for 15 min. Super Q traps were cleaned by soxhlet extraction with methylene chloride for 24 h and dried in a fume hood prior to each use. Volatile chemicals were eluted from the Super Q adsorbent with 200 µl of high purity methylene chloride (99.5% pure; ACROS, Morris Plains, NJ, USA). An aliquot of C_16_ standard (5 µg) was added to each sample for quantitative analysis.

Chemical extracts were analyzed by using gas chromatography (ThermoQuest Trace GC 2000, Austin, TX, USA). The column was fused silica, 25 m long, 0.25 mm i.d., DB-5MS phase (J & W Scientific, Agilent Technologies, Santa Clara, CA, USA), programmed from 50 to 130°C at 15.0°C/min, then from 130 to 220°C at 10.0°C/min, and then held at 220°C for 4 min. The column used in the gas chromatograph interface to the mass spectrometer (Agilent Technologies 5975B) was 25 m long, 0.25 mm i.d., DB-5MS phase (J & W Scientific, Agilent Technologies), programmed at 40°C for 2 min, then from 40 to 130°C at 10.0°C/min, then from 130 to 220°C at 20.0°C/min, and then held at 220°C for 4 min. Chemicals were identified by using the NIST mass spectral program (version 2.0 d) and the NIST/EPA/NIH mass spectral library (NIST11) when Reverse Matches and Matches were >950 and >900‰, respectively. Identifications were then verified by comparing the mean Kovats Retention Index (RI) with the RI calculated from synthetic chemicals, when commercially available [RI = 1045, 1358, 1391, 1443, 1477, 1532 for eucalyptol (≥99.0%; Fluka Analytical, Steinheim, Germany), (−)-α-cubebene (≥97.0%; Bedoukian Research Inc., Danbury, CT, USA), (−)-α-copaene (≥90.0%; Fluka Analytical), (−)-β-caryophyllene (≥98.5%; Sigma Chemical Co., St. Louis, MO, USA), α-humulene (≥96.0%; Sigma Chemical Co.), and (+)-δ-cadinene (≥97.0%; Fluka Chemie, Buchs, Switzerland), respectively]. Alternatively, experimental RI values were compared with previously published data (δ-elemene [48], β-elemene [49]–[50], and calamenene [48], [51]).

### Electroantennography

EAG substrates consisted of: ethanol (5 ml ethyl alcohol 95%; Pharmco-Aaper, Brookfield, CT, USA); a manuka oil lure (the standard lure for *X. glabratus* [31], release rate 50 mg oil per day; Synergy Semiochemicals Corp.); silkbay shavings (15 g freshly-rasped bark and cambium, collected at ABS, prepared as reported previously [45]); and the synthetic terpenoids eucalyptol, α-cubebene, α-copaene, β-caryophyllene, α-humulene, and δ-cadinene (each 50 µl neat oil; obtained from the suppliers identified above). Each substrate was placed into a separate 250 ml hermetic glass bottle equipped with a lid that had been fitted with a short thru-hull port (Swagelok, Solon, OH, USA) and silicone septum (Alltech, Deerfield, IL, USA). Sample bottles were sealed and equilibrated for 2 h at 24°C to allow for headspace saturation with volatiles.

**Figure 1:**
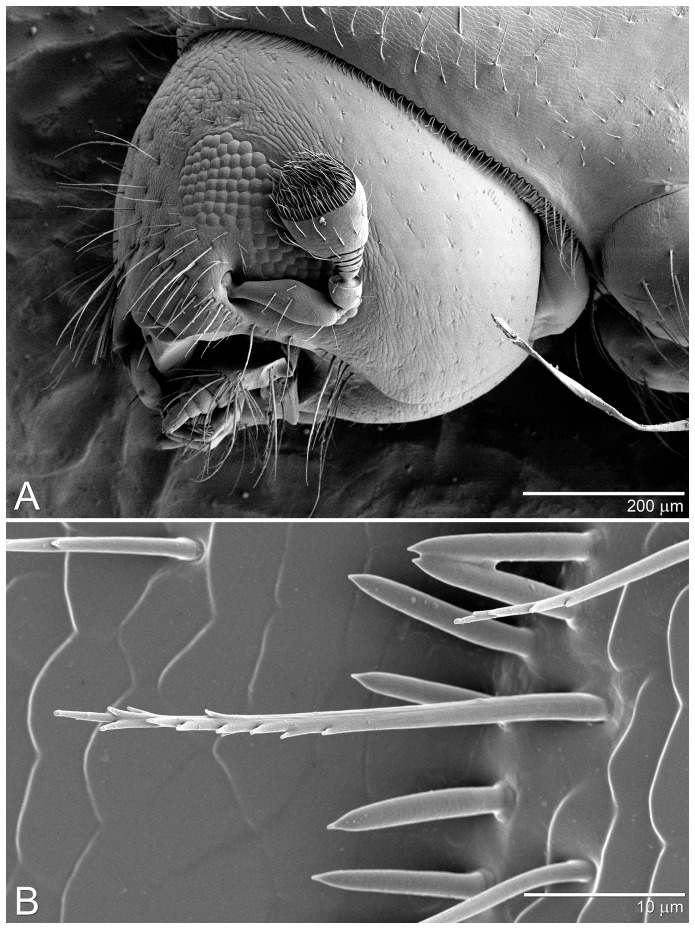
Scanning electron micrographs of adult female *Xyleborus glabratus*. (A) Full view of head showing intact left antenna; the apical ventral surface of antennal club is flattened and bears concentric arrays of sensilla. (B) Detail of antennal club reveals two types of sensilla: long tapered sensilla trichoidea (which bear minor branching morphology at the distal end), and more numerous short bluntly-pointed sensilla basiconica. Antennal preparations for electrophysiological recordings were mounted ventral surface facing upwards, with electrode contact on the dorsum of the club to avoid coating olfactory sensilla with conductive gel.

Instrumentation consisted of a Syntech EAG system (Syntech Original Research Instruments, Hilversum, Netherlands), which included a micromanipulator assembly (MP-15), a data acquisition interface box (serial IDAC-232), a stimulus air controller (CS-05), and EAG 2000 software. Olfactory responses were recorded with a newly developed technique [33] which utilized a gold-plated 2-pronged antennal holder (Syntech EAG Combi-Probe) modified with thin gold wire to accommodate the minute antennae of *X. glabratus* (mean antennal length 0.37±0.01 mm). Single excised antennae were mounted, ventral side facing up, between electrodes using salt-free gel (Spectra 360, Parker Laboratories, Fairfield, NJ, USA). LT-SEM revealed that the ventral surface at the apex of the antennal club is flattened and bears a dense array of concentrically arranged olfactory sensilla (Fig. 1); thus, care was taken to not coat this region with conductive gel.

A stream of humidified air, purified with activated charcoal granules (grain size 1–2 mm), was passed continuously over the antennal preparation at 400 ml/min. The tip of the delivery tube was placed ∼1 mm from the antenna, and the air controller was configured to allow for pulse flow compensation during sample delivery. Using gas tight syringes (SGE Analytical Science, Victoria, Australia), samples of saturated vapor were withdrawn from the test bottles, injected into the airstream, and presented to the antennae. In each recording session, the antenna was presented first with ethanol (2 ml saturated vapor), which has been shown previously to serve as an appropriate standard and positive control for *Xyleborus* species [33]. This was followed by injection of test samples in random order, then with negative controls consisting of clean air injections equal in volume to the sample injections, and ended with a final injection of ethanol. There was a 2 min interval (clean air flush) between sample injections to prevent antennal adaptation (diminished EAG response as a result of repeated exposure to specific chemical stimuli).

EAG responses to test substrates were measured initially in millivolts (peak height of depolarization) and then normalized to percentages relative to the EAG response obtained with ethanol. Normalization with a standard reference chemical corrects for time-dependent variability (gradual decline) in antennal performance, and also allows for comparison of relative EAG responses obtained with different substrates [45], [52] and with different cohorts of insects [53]. Finally, any response recorded with the negative control was subtracted from the normalized test responses to correct for ‘pressure shock’ caused by injection volume. All statistical analyses were performed using the corrected normalized EAG values.

Two EAG experiments were conducted with host-based attractants. The first experiment was designed to evaluate dose-dependent EAG responses of female *X. glabratus* to various terpenoid compounds. Six doses, in a two-fold series of headspace volumes ranging from 0.25 to 6.0 ml, were used to quantify antennal response to volatiles emitted from three test substrates: silkbay shavings, manuka oil lure, and synthetic α-copaene. Based on the dose-response results obtained in this initial experiment, a second experiment was conducted using fixed 2 ml doses to compare EAG responses to silkbay wood, manuka lure, and all six synthetic terpenoids. To construct dose-response curves, EAG responses were recorded from antennae of 10–15 replicate females for each substrate; for the comparative EAG experiment, responses were measured from 15–20 replicate females.

### Statistical Analysis

Analyses of variance (ANOVA) (Proc GLM, SAS Institute [54]) were conducted for results from the field tests and comparative EAG experiments, followed by mean separations with Tukey test (*P*<0.05). The Box-Cox procedure, which is a power transformation that regresses log-transformed standard deviations (*y*+1) against log-transformed means (*x*+1), was used to determine the type of transformation necessary to stabilize variance prior to analysis [55]. Regression analysis (Systat Software [56]) was used to describe the relationships between substrate dose and EAG responses (with separate analyses for each substrate), and also to document temporal patterns in boring behaviors observed in the no-choice laboratory bioassays. Analysis by *t*-test [56] was performed to measure differences between EAG responses to equal doses of two different substrates, and differences between responses to adjacent doses of the same substrate. For each sesquiterpene and eucalyptol, the captures of *X. glabratus* in field test 1 were compared to the quantity of chemical emitted per substrate (10 replicate bolts per tree species) by using Pearson product moment correlation [56].

## Results

### Field Tests

**Figure 2:**
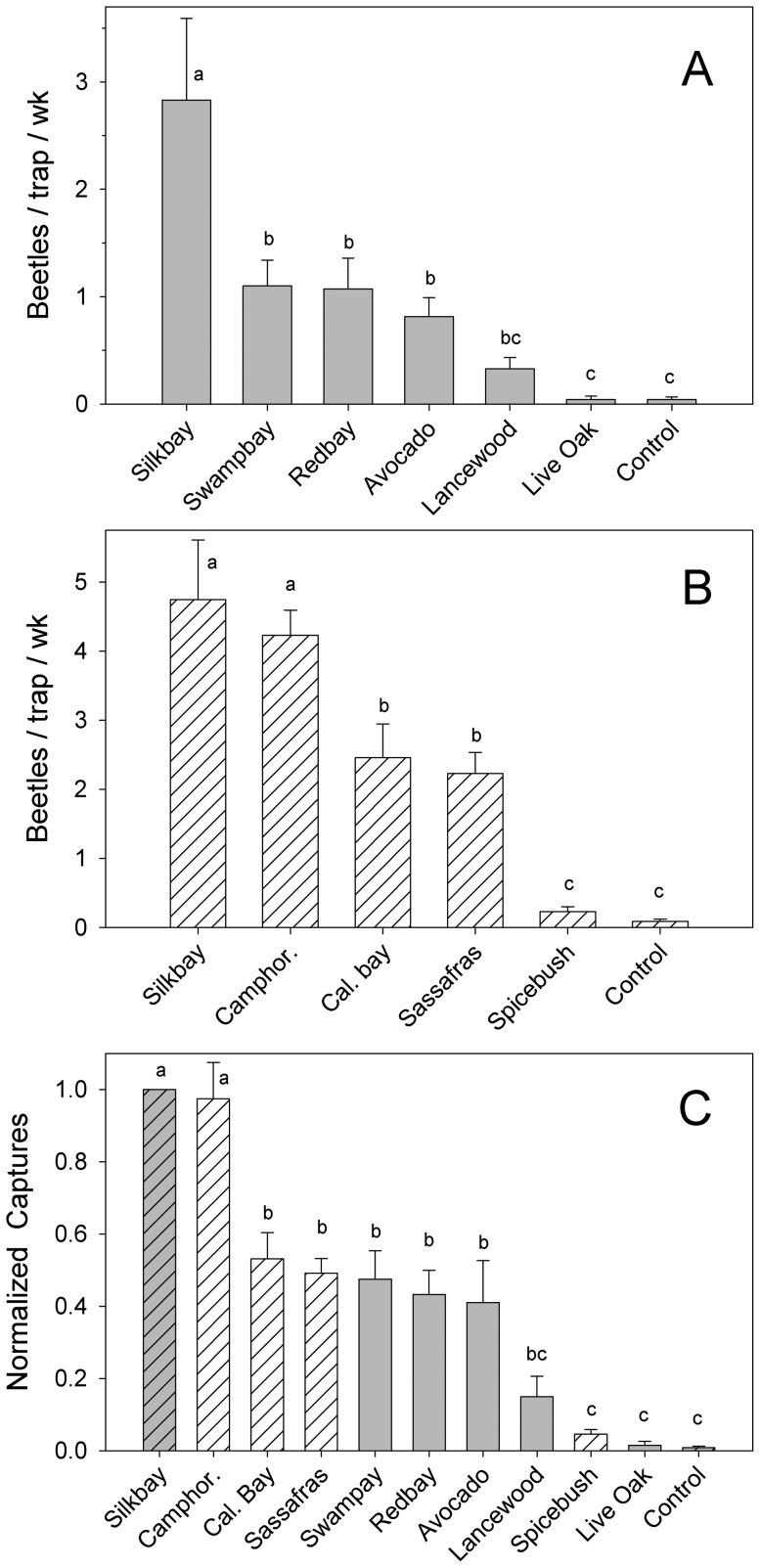
Mean (± SE) captures of female *Xyleborus glabratus* in field tests conducted in Florida, USA. (A) Test 1 evaluated captures in sticky traps baited with bolts of silkbay *Persea humilis*, swampbay *P. palustris*, redbay *P. borbonia*, avocado *P. americana*, lancewood *Nectandra coriaceae*, and live oak *Quercus virginiana*. (B) Test 2 evaluated captures with silkbay (for comparison with test 1), camphor tree *Cinnamomum camphora*, California bay laurel *Umbellularia californica*, sassafras *Sassafras albidum*, and northern spicebush *Lindera benzoin*. Both tests included an unbaited control trap. (C) To estimate relative attraction among all Lauraceae, the results of tests 1 and 2 have been normalized and combined; normalization consisted of expressing captures as a percentage relative to silkbay (the most attractive treatment in both tests). Bars topped with the same letter are not significantly different (Tukey mean separation of square root [x+0.5]-transformed data, non-transformed means presented, P<0.05).

**Figure 3:**
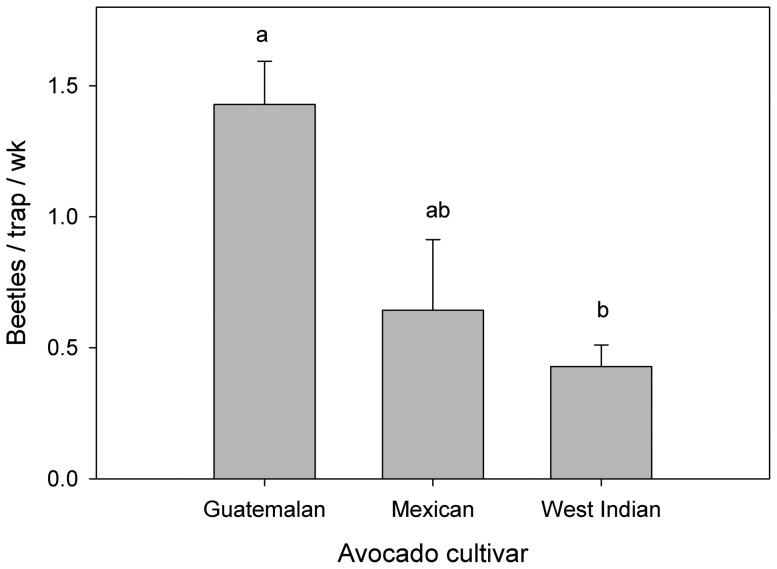
Mean (± SE) captures of female *Xyleborus glabratus* with avocado cultivars in field test 1. Varieties tested included ‘Taylor’ (Guatemalan race), ‘Duke’ (Mexican race), and ‘Catalina’ (West Indian race). Bars topped with the same letter are not significantly different (Tukey mean separation, P<0.05).

In field test 1 (Fig. 2A, Table S1), there were differences in mean capture of *X. glabratus* among the seven treatments (*F* = 14.58; df = 6, 63; *P*<0.0001). Traps baited with bolts of silkbay caught significantly more beetles than any other treatment. Traps baited with swampbay, redbay, or avocado (all three cultivars combined) caught comparable numbers of beetles, which were significantly higher than numbers caught with live oak or the unbaited trap. Captures with lancewood were the lowest observed among the Lauraceae treatments in test 1, with results intermediate between those obtained with known hosts (swampbay, redbay, avocado) and the non-host control (oak). When the results with avocado were analyzed separately (Fig. 3), there were differences in mean captures among the three varieties (*F* = 5.49; df = 2, 7; *P* = 0.037). Captures with the Guatemalan cultivar ‘Taylor’ were significantly higher than those obtained with the West Indian cultivar ‘Catalina’. Captures with the Mexican cultivar ‘Duke’ were intermediate.

Overall, numbers of *X. glabratus* were higher during the second field test (Fig. 2B, Table S2), and there were differences in mean captures among the six treatments (*F* = 32.32; df = 5, 24; *P*<0.0001). As observed in test 1, highest captures were obtained in traps baited with silkbay bolts; however, captures obtained with camphor tree were not significantly different. Traps baited with either California bay laurel or Sassafras had the next highest captures. Captures with spicebush were very low, and not statistically different from those obtained with the unbaited control.

Since the population levels were different at the two sites used for field testing, captures of *X. glabratus* were normalized to facilitate comparison. Normalization consisted of converting raw numerical captures to percentages relative to the captures obtained with silkbay, the most attractive treatment in each field test. The combined normalized data are presented in Fig. 2C, to provide an estimate of relative attraction for all ten tree species evaluated in the study.

### Laboratory Bioassays

**Figure 4:**
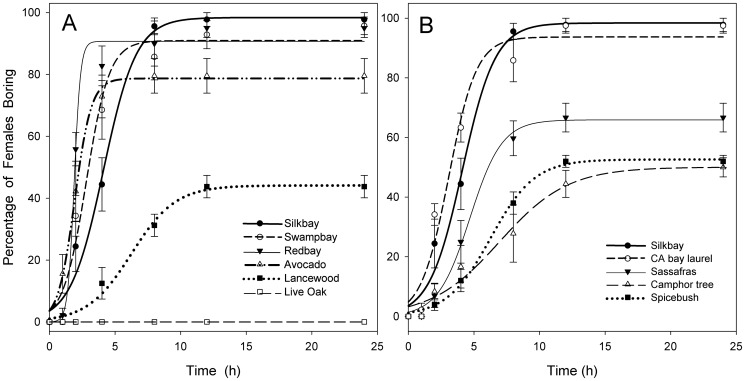
Mean (± SE) percentage of female *Xyleborus glabratus* boring into bolts in 24 hr bioassay. Each tree species was evaluated separately in no-choice tests, but to facilitate comparison, results are grouped according to treatment deployment in field test 1 (A) or field test 2 (B). Rate of boring with all species of Lauraceae was best fit by regression analysis with sigmoidal models (see Table 1).

Composite results of the no-choice bioassays are presented in Fig. 4 (and Table S3); for comparative purposes, results are grouped according to treatment deployment in field test 1 (Fig. 4A) or field test 2 (Fig. 4B). Boring was observed on bolts from all nine species of Lauraceae, and regression analysis with sigmoidal models (sigmoid, three parameter models) best described the relationships between the time after bolt presentation and the percentage of females actively boring (Table 1). The sigmoidal equation is expressed in the form: *y* = a/(1+*e* ^−[(x-b)/c]^), where *x* represents time (h), *y* represents boring response (%), coefficient ‘a’ represents the maximum boring response, and coefficients ‘b’ and ‘c’ reflect the rate at which maximum response is attained [29]. Boring was initiated most quickly on bolts of the four *Persea* species (Fig. 4A) and California bay laurel (Fig. 4B), and maximum percentages were achieved within 4 to 8 h. In contrast, on bolts of lancewood (Fig. 4A), camphor tree, and spicebush (Fig. 4B), females spent considerably more time walking over the substrate before selecting a site and committing to boring activity. This resulted in a considerable lag time (relative to *Persea* and bay laurel) in boring response, and maximum percentages were not reached until 12 to 15 h. On bolts of sassafras, rates of boring were intermediate between those for the two former groups (Fig. 4B). Few beetles responded to the bolts of live oak (Fig. 4A). Although most continued to wander throughout the test arena, several females settled into natural crevices or under the bark at the cut ends of the oak bolts. This behavior was interpreted as a thigmotactic response and not boring.

**Table 1:** Analysis of boring response of female *Xyleborus glabratus* presented with wood bolts from nine species of North American Lauraceae in a 24 hour no-choice bioassay (N≥5 per species).

	Regression		Final boring percentage	Percentage boring on cut surface
Species	Equation	*R* ^2^	(Mean ± SE)^1^	(Mean ± SE)^1^
Silkbay	*y* = 98.28/(1+*e* ^-[(x-4.11)/1.44]^)	0.991	97.8±2.2 a	97.5±2.5 a
California bay laurel	*y* = 93.70/(1+*e* ^-[(x-3.09)/1.06]^)	0.974	97.5±2.5 a	86.0±6.0 ab
Swampbay	*y* = 90.94/(1+*e* ^-[(x-2.82)/0.88]^)	0.976	95.7±2.9 a	83.6±5.5 ab
Redbay	*y* = 90.70/(1+*e* ^-[(x-1.89)/0.24]^)	0.991	95.0±3.1 a	90.5±2.5 ab
Avocado	*y* = 78.72/(1+*e* ^-[(x-1.93)/0.64]^)	0.996	79.6±5.6 b	79.2±4.1 b
Sassafras	*y* = 65.92/(1+*e* ^-[(x-4.69)/1.22]^)	0.997	66.7±4.8 c	84.3±6.7 ab
Spicebush	*y* = 52.62/(1+*e* ^-[(x-6.28)/1.67]^)	0.997	52.0±2.0 cd	59.7±7.5 c
Camphor tree	*y* = 50.05/(1+*e* ^-[(x-6.95)/2.60]^)	0.976	50.0±3.2 d	85.0±9.6 ab
Lancewood	*y* = 44.12/(1+*e* ^-[(x-6.25)/1.68]^)	0.988	43.8±3.6 d	100±0.0 a

1 Means followed by the same letter within a column are not significantly different (Tukey mean separation, *P*<0.05).

After 24 h, there were significant differences among the ten treatments (*F* = 79.76; df = 9, 49; *P*<0.001). Mean separation analysis distinguished four groupings within the Lauraceae, with the highest final percentages on 1) silkbay, California bay laurel, swampbay, and redbay, followed by 2) avocado, 3) sassafras and spicebush, and 4) spicebush, camphor tree, and lancewood (Table 1). Most of the boring on these species occurred on the cut ends of the bolts rather than through the bark, but there were differences among treatments (*F* = 3.99; df = 8, 48; *P* = 0.001). When data for avocado were analyzed separately, there were no differences among the three cultivars (*F* = 0.46; df = 2, 12; *P* = 0.639); thus, results for avocado were pooled in Fig. 4 and Table 1.

### Chemical Analysis

**Figure 5:**
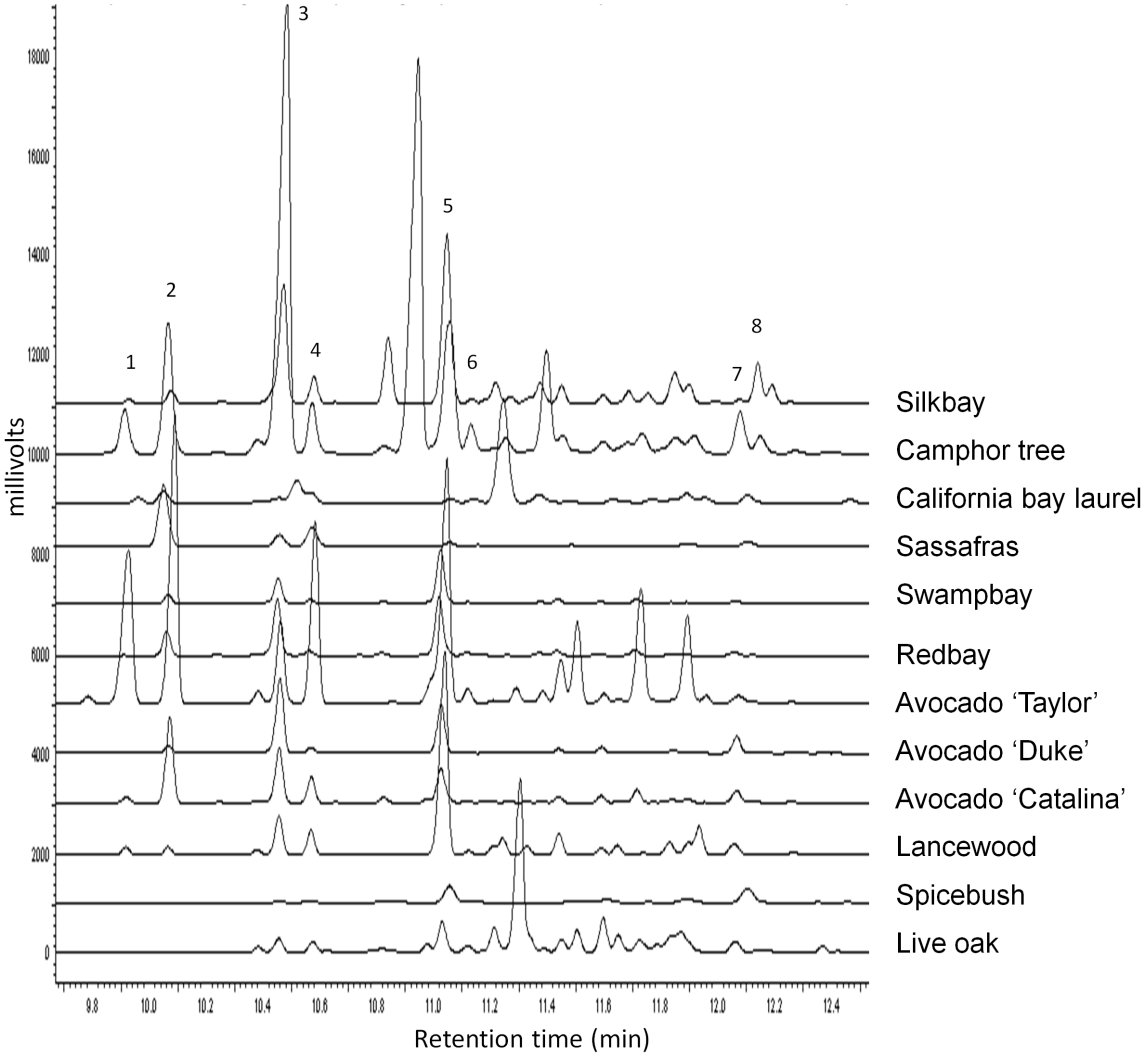
Representative chromatographic analyses of sesquiterpenes from North American Lauraceae. Volatiles were isolated from 6-MS (DB-5MS column). Peak identifications are as follows: 1 = δ-elemene, 2 = α-cubebene, 3 = α-copaene, 4 = β-elemene, 5 = β-caryophyllene, 6 = α-humulene, 7 = δ-cadinene, 8 = calamenene.

A total of 72 volatile chemicals (detected at quantities ≥0.5 µg in at least one sample) were isolated by Super Q collections and GC-MS analysis. Of these, there were eight sesquiterpenes common among the Lauraceae (Table 2, Table S4). Sesquiterpene content varied both qualitatively and quantitatively among the species (Fig. 5), but only four chemicals were positively correlated with captures of *X. glabratus* in field tests: α-cubebene (Pearson correlation coefficient = 0.243, *P* = 0.042; Fig. 5 peak 2), α-copaene (coefficient = 0.553, *P*<0.0001; Fig. 5 peak 3), α-humulene (coefficient = 0.299, *P* = 0.015; Fig. 5 peak 6), and calamenene (coefficient = 0.465, *P*<0.0001; Fig. 5 peak 8).

**Table 2:** Mean (±SE) quantity (µg) of volatile terpenoids emitted from wood bolts of North American Lauraceae.

	eucalyptol^2^	δ-elemene^3^	α-cubebene^2^	α-copaene^2^	β-elemene^3^	β-caryophyllene^2^	α-humulene^2^	δ-cadinene^2^	calamenene^3^
RI^1^	1045	1343	1357	1390	1400	1443	1478	1530	1536
Silkbay	47.7±11.6	0.3±0.1	3.5±0.9	9.1±1.7	1.8±0.5	16.6±4.3	1.6±0.3	0.7±0.1	3.2±0.7
Camphor tree	7.6±3.5	4.3±1.9	9.6±1.9	23.0±5.5	3.3±0.7	10.4±2.1	0.6±0.2	2.4±0.3	0.8±0.2
Cal. bay laurel	396.5±36.9	0.0±0.0	1.6±0.1	0.9±0.1	1.3±0.1	0.7±0.1	0.5±0.0	1.3±0.1	0.0±0.0
Sassafras	2.0±0.6	0.0±0.0	6.1±1.7	1.3±0.3	2.0±0.5	0.6±0.2	0.2±0.1	0.6±0.1	0.1±0.0
Swampbay	60.1±10.1	0.1±0.0	0.8±0.2	5.1±1.3	0.3±0.1	4.5±1.1	0.4±0.1	0.5±0.1	1.0±0.0
Redbay	145.1±31.6	0.0±0.0	1.0±0.3	5.0±3.0	0.7±0.3	5.4±1.3	0.5±0.1	0.8±0.3	1.0±0.0
Avocado-Guat.	6.4±3.5	20.0±9.5	35.1±17.6	9.3±4.3	19.2±9.0	28.5±12.6	8.2±2.9	1.6±0.7	0.0±0.0
Avocado-Mex.	0.0±0.0	0.2±0.0	1.1±0.1	9.6±0.3	0.9±0.0	7.9±0.6	0.1±0.0	2.5±0.1	0.3±0.0
Avocado-W.Ind.	0.0±0.0	1.2±0.2	11.5±1.4	8.1±1.0	3.5±0.3	5.5±0.5	0.1±0.0	2.4±0.2	0.5±0.0
Lancewood	0.0±0.0	1.6±0.4	0.8±0.1	3.6±0.6	2.7±0.4	14.6±3.8	0.3±0.0	1.3±0.3	0.3±0.0
Spicebush	72.1±14.9	0.0±0.0	0.1±0.0	0.2±0.0	0.5±0.1	3.5±0.6	0.4±0.1	4.2±0.7	0.1±0.0
Live oak	0.1±0.0	0.0±0.0	0.1±0.0	1.0±0.3	1.30±0.2	4.2±0.6	0.6±0.1	0.9±0.1	0.3±0.0

Volatiles were isolated from 6 g samples of rasped bark and cambium by super Q collection, and then analyzed by GC-MS (DB-5MS column). Avocado cultivars tested included ‘Taylor’ (Guatemalan race), ‘Duke’ (Mexican race), and ‘Catalina’ (West Indian race).

1 Mean Kovats Retention Index calculated from 3–10 replicate bolts per species.

2 Identification by NIST/EPA/NIH mass spectral library (NIST11), then verified by comparison with RI from synthetic chemicals (see text).

3 Identification by NIST/EPA/NIH mass spectral library (NIST11), then verified by comparison with RI from published reports (see text).

The volatile profile from camphor tree, one of the most attractive species in the field (Fig. 2B) but with one of the lowest boring percentages in bioassays (Fig. 4B), contained large amounts of α-copaene and α-cubebene, but also contained a large sesquiterpene peak (RI = 1437) not detected in other Lauraceae (tentative NIST library identification as β-santalene). The Guatemalan avocado ‘Taylor’, the most attractive cultivar tested (Fig. 3), contained significantly higher quantities of many sesquiterpenes, including δ-elemene, α-cubebene, β-elemene, β-caryophyllene, and α-humulene (Table 2). ‘Taylor’ also had detectable levels of eucalyptol, not seen in the two other avocado cultivars. However, eucalyptol content was highly variable among species of Lauraceae. It was found at very high levels in California bay laurel and redbay, at relatively low levels in attractive species like camphor tree and sassafras, and at moderate levels in unattractive species like spicebush (Table 2, Table S4); consequently, eucalyptol was not correlated with captures of *X. glabratus* in the field (coefficient = 0.078, *P* = 0.520).

### Electroantennography

**Figure 6:**
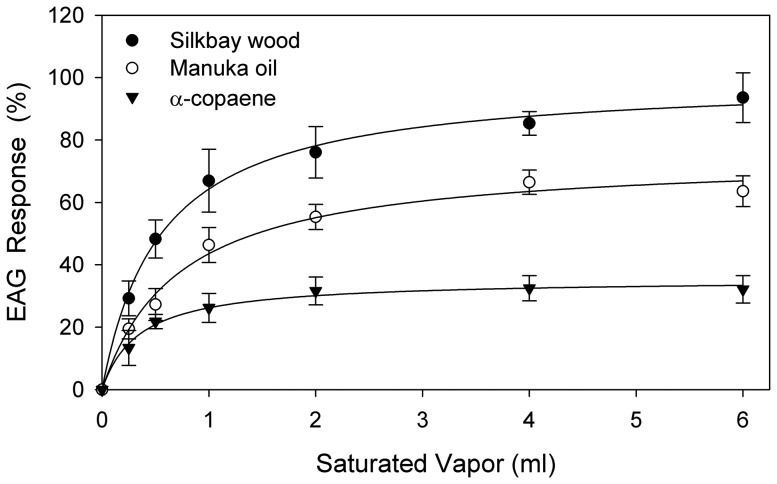
Electroantennogram dose-response profiles constructed from mean (± SE) antennal responses of female *Xyleborus glabratus*. Test substrates included freshly-rasped wood of silkbay *Persea humilis*, a commercial manuka oil lure, and synthetic α-copaene. Responses are expressed as normalized percentages relative to a standard reference compound (ethanol, 2 ml saturated vapor). Dose-response curves generated with hyperbolic regression models (see text).

The relationships between doses of volatile chemicals and amplitudes of EAG responses (Fig. 6, Table S5) were best fit by regression with hyperbolic models (single rectangular, two parameter models). The general equation is expressed in the form: *y* = a*x*/(b+*x*), where *x* represents the substrate dose (ml), *y* represents the normalized EAG response (%), and the coefficients ‘a’ and ‘b’ represent maximum EAG response and receptor binding affinity, respectively. Hyperbolic equations are used frequently for ligand-binding studies, and have been shown previously to serve well for characterization of EAG dose-response relationships [45], [52]. The EAG regression equations were as follows: silkbay wood: *y* = 99.75*x*/(0.53+*x*), *R* ^2^ = 0.996; manuka oil lure: *y* = 77.44*x*/(0.74+*x*), *R* ^2^ = 0.988; and α-copaene: *y* = 36.76*x*/(0.40+*x*), *R* ^2^ = 0.995. With all substrates, EAG amplitude increased with dosage up through approximately 2 ml, and then reached a plateau. At doses ≤1 ml, there was not consistent separation among mean responses recorded with the three substrates. For example, at 0.5 ml, there was no difference between responses obtained with manuka oil and α-copaene (*t* = −1.164, df = 28, *P* = 0.254), and at 1.0 ml, there was no difference between responses obtained with manuka oil and silkbay wood (*t* = 1.861, df = 28, *P* = 0.073). When dosages were increased to 2 ml, there were significant differences in EAG response among the treatments (*F* = 14.186; df = 2,42; *P*<0.001); response elicited with silkbay wood was higher than that with manuka oil, and response elicited with manuka oil was higher than that with α-copaene.

**Figure 7:**
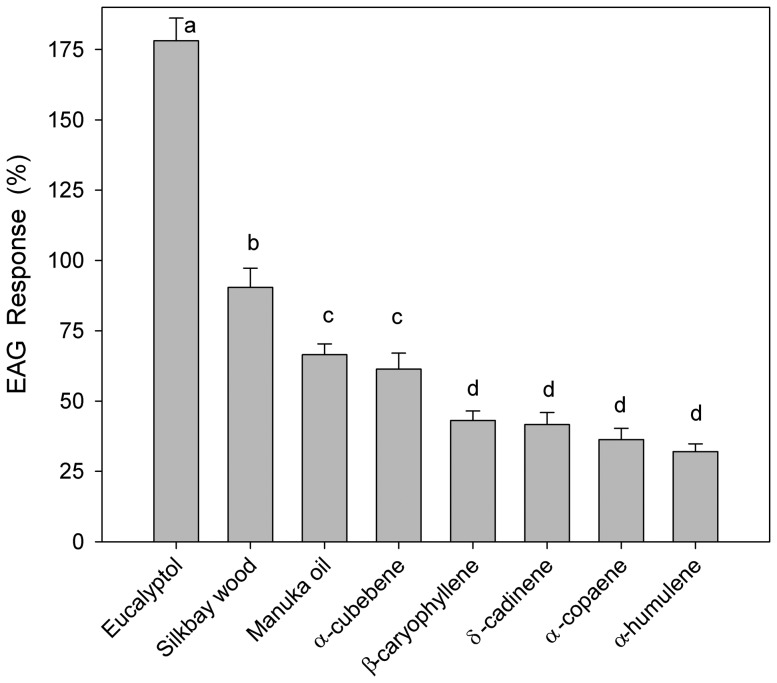
Mean (± SE) electroantennogram responses of female *Xyleborus glabratus* to host-based volatiles (2 ml doses). Test substrates included freshly-rasped wood of silkbay *Persea humilis*, a commercial manuka oil lure, and six synthetic terpenoids. Responses are expressed as normalized percentages relative to a standard reference compound (ethanol, 2 ml saturated vapor). Bars topped with the same letter are not significantly different (Tukey mean separation of square root [x+0.5]-transformed data, non-transformed means presented, P<0.05).

There was no significant increase in EAG response when doses of the test substrates were increased to 4 ml or 6 ml (Fig. 6). Thus, 2 ml doses were assumed to saturate the olfactory receptors of the antennae, and fixed 2 ml doses were used for the comparative EAG experiment (Fig. 7, Table S6). There were significant differences in antennal response elicited with the eight test substrates (*F* = 58.153; df = 7,133; *P*<0.001), and mean separation analysis identified four groupings. The highest amplitude response was obtained with 1) eucalyptol, and the next highest response with 2) silkbay shavings, followed by 3) manuka oil lure and α-cubebene, and 4) the other four sesquiterpenes: β-caryophyllene, δ-cadinene, α-copaene, and α-humulene.

## Discussion

Complex interactions underlie the epidemiology of laurel wilt in forest and agricultural ecosystems. Although root grafting between adjacent trees accelerates spread of the pathogen in affected areas, especially where there are high densities of host trees (e.g. commercial avocado plantings), initial pathogen transmission and disease expression require an intimate association among three species – an insect vector (female *X. glabratus*, and potentially other species), a pathogenic fungal symbiont (*R. lauricola*), and a woody host tree (New World Lauraceae) that is attractive to the vector, supports growth of the symbiont, and recognizes the fungus as foreign (by mechanisms yet unknown) to induce systemic defensive responses. The present investigation focused on the initial steps of this process – host location and recognition by a foundress *X. glabratus*. There were two main objectives of the comparative study. First, by assessing relative attraction and boring preferences within the Lauraceae, we sought to identify the species that were most susceptible to attack by *X. glabratus*. Second, by relating behavioral responses with volatile emissions from test substrates, we sought to gain an enhanced understanding of the semiochemicals used by *X. glabratus* for host-location. These two objectives will be discussed separately.

### Susceptibility to Attack by *X. glabratus*

Based on the high relative attraction in the field and the high percentages of individuals that exhibited boring behavior in bioassays (≥95%), the species that were most vulnerable to attack were silkbay, swampbay, redbay, and California bay laurel. These results are consistent with observations of native *Persea* species in the southeastern U.S. to which this highly efficient vector has transmitted *R. lauricola*. In the Atlantic Coastal Plain communities of Georgia and South Carolina, redbay and swampbay populations frequently experience mortality in excess of 90 percent within two years of the onset of laurel wilt [1]. With the more recent spread of laurel wilt into south-central Florida, stands of silkbay are beginning to die off in the dry scrub habitats along the Lake Wales Ridge ecosystem [33]. Although breeding populations of *X. glabratus* have not been detected west of Mississippi, future expansion of the pest range could have a severe negative effect on California bay laurel, which is a significant component of Pacific Coastal forests in California and Oregon. The strong attraction and boring behaviors that were observed with this species in the present study corroborate results from parallel tests conducted in South Carolina [25]. Notably, that latter study demonstrated that California bay laurel is not only attractive to *X. glabratus*, but is also a suitable reproductive host; and previous work indicated that the species is susceptible to laurel wilt after artificial inoculations of *R. lauricola* [57].

The present results indicated that avocado and sassafras are less vulnerable to attack by *X. glabratus* than the above species. Both species were as attractive as swampbay, redbay, and California bay laurel in the field trial, but exhibited lower rates of boring activity in the laboratory bioassay (80% and 67%, respectively). At a field site in South Carolina, the numbers of *X. glabratus* entrance holes were significantly lower on sassafras than on swampbay bolts in 2010 [26], but were significantly higher on sassafras bolts than on swampbay in 2011 [25]. A possible explanation for these seemingly conflicting results is that severe depletion of swampbay trees between 2010 and 2011 may have led to the selection of beetles that could successfully colonize a ‘less preferred’ host. Alternatively, there may be genetic variation in the attractiveness of these native trees to *X. glabratus*.

There is variation among the cultivated avocado varieties that have been examined. There was no difference in attraction between avocado and four other tree species when results from all avocado treatments were combined in the present study. However, individual assessments indicated that the Guatemalan cv. ‘Taylor’ was significantly more attractive than the other two cultivars, and this difference was associated with much higher terpenoid emissions in cv. ‘Taylor’ (discussed below). Previously, more *X. glabratus* were caught with a Guatemalan cv., ‘Brooks Late’, than with West Indian (cv. ‘Simmonds’) or Mexican (cv. ‘Seedless Mexican’) genotypes [29]. Although these numerical differences were not statistically significant, GC-MS analysis indicated that ‘Brooks Late’ had significantly higher sesquiterpene emissions than the other two cultivars [29].

Additional evaluations are needed to determine if trees of the Guatemalan race are, in general, more attractive to dispersing *X. glabratus* than trees from the other two lineages. Clearly, these results have implications for breeding programs that would develop laurel wilt tolerant cultivars of this important crop. Although avocado appears to be less suitable as a reproductive host for *X. glabratus* than U.S. native *Persea* species [58]–[59], beetle reproduction is not required for transmission of *R. lauricola*, only host recognition and boring. In laboratory bioassays, percentage of boring was equivalent among the cultivars compared in this study and among those compared previously [29]. More information is needed on the transmission of this pathogen to avocado and other host species by *X. glabratus* and other potential vector species [9], [16].

The remaining species in the Lauraceae that were tested – camphor tree, lancewood, and northern spicebush – are apparently less vulnerable to attack by *X. glabratus*. Despite attracting high numbers of *X. glabratus* in the field test, relatively low boring activity was observed on camphor tree in the laboratory bioassay. Low boring incidences were also observed on lancewood and spicebush; in addition, field captures with these treatments were no different from those obtained with unbaited traps and non-host control traps.

### Host Location and Acceptance

The dispersal period for ambrosia beetles is a brief but critical stage in their life. Females engage in flight to locate and colonize new resources necessary for reproduction, but this exposes them to potential predation and harsh environmental conditions. Thus, it would be highly adaptive for females to have efficient host-seeking behaviors (guided by reliable cues), coupled with appropriate timing to minimize risks. The location, recognition, and final acceptance of a host can be viewed as a multi-step process that requires a series of cues presented in sequential order. Based on current evidence, the following scenario is proposed.

Initiation of flight activity is determined by an interaction of environmental cues, predominantly light intensity, temperature, and relative humidity [60]. Temporal flight patterns are species-specific, with females of *X. glabratus* having peak dispersal during the late afternoon and early evening hours [33], [42]. After departing the natal tree, females (usually sibling mated) orient toward long-range olfactory cues while in flight. It does not appear that *X. glabratus* utilizes sex or aggregation pheromones, since bolts from trees infested with conspecifics are no more attractive than bolts from uninfested trees [2]. As primary colonizers of healthy trees [1], females are attracted to natural volatiles emitted by host Lauraceae [29], [31], and the current study identified four sesquiterpenes that were correlated with attraction: α-cubebene, α-copaene, α-humulene, and calamenene. Of those, α-cubebene and α-copaene are the two major components found in attractive hosts and also in attractive essential oils, particularly cubeb oil [48], recently identified as the most effective lure for detection of *X. glabratus* [21], [35]. α-Cubebene also elicited the highest EAG response of the pure sesquiterpenes that were tested (comparable to EAG response to manuka oil, a mixture of many terpenoid compounds); thus, α-cubebene may be a stronger attractant than α-copaene.

In contrast, eucalyptol appears to be the key attractant emitted from California bay laurel. Eucalyptol alone, in high doses, has been shown to attract *X. glabratus* [39], and bay laurel was unusual in that eucalyptol was the principal component in its suite of volatiles, which was particularly low in sesquiterpenes. Eucalyptol emissions were also high from redbay, the species that facilitated establishment of *X. glabratus* in North America [1]–[2], and the volatile ether evoked a strong EAG response, significantly higher than the sesquiterpenes tested. However, the higher EAG response recorded with eucalyptol may be the result of differences in volatility (vapor concentration) between the monoterpene (C_10_) and the 50% higher molecular weight sesquiterpenes (C_15_) [61], and not necessarily an indication of differences in number of antennal receptors. None the less, eucalyptol warrants further evaluation as a long-range attractant for *X. glabratus* and potential economical field lure. Interestingly, eucalyptol is lacking or present in only trace amounts in attractive essential oils (e.g. cubeb [48], manuka [62]). This information supports the hypothesis that multiple chemical cues (either in combination or alternatively) may contribute to the host location process of *X. glabratus*.

As females approach the source of long-range attractants, mid-range visual cues are likely assessed to direct flight toward individual trees and locations on a given tree. Since the flight window of *X. glabratus* occurs several hours earlier than most other Scolytinae in the southeastern U.S., including non-pest *Xyleborus* species [33], [43], *X. glabratus* may rely on visual cues more than other ambrosia beetles. Field surveys indicate that the oldest, largest-diameter trees are typically the first to be attacked by *X. glabratus* and succumb to laurel wilt, and beetle entrance holes are more numerous on the trunk and large diameter branches [1], [15]. This preference would be adaptive, as larger diameter hosts would support more extensive gallery formation and increase the reproductive potential (fitness) of *X. glabratus*, as has been shown for other species of ambrosia beetle [63]–[64].

A recent study demonstrated experimentally that females indeed utilize stem diameter as a host-seeking cue [41], but this visual signal only synergizes attraction when in the proper chemical context. Thus, *X. glabratus* does not bore into trunks/branches of suitable diameter if they lack the appropriate chemical cues (e.g. oak in the present study). Our recent analysis of terpenoid distributions throughout avocado trees again suggests that α-cubebene and α-copaene are important reference components of that chemical context [38]. In avocado, both sesquiterpenes follow a concentration gradient along a proximo-distal axis from trunk, through branches to leaves, with the highest emissions measured from the trunk. This chemical gradient, in combination with visual cues, could be used as a reliable feature with which optimal sites for landing and subsequent initiation of reproductive effort could be identified on potential host trees.

Although little is known about the short-range cues that trigger a behavioral switch from host-seeking to host-acceptance and boring, it probably involves a complex integration of multiple signals once females contact potential hosts. These may include olfactory, gustatory, contact chemosensory, tactile, and visual stimuli, all of which must reinforce the message that a suitable host has been located (i.e. the substrate must smell, taste, feel, and look ‘right’ before females commit to boring). For example, in addition to volatile host terpenoids, secondary olfactory cues may be detected by the antennae, including fungal odors if a tree is already infested with ambrosia beetles.

It has been demonstrated in short-range bioassays that *X. glabratus* is attracted to volatiles emitted from *R. lauricola* as well as symbionts from other ambrosia beetles [65]; and a blend of volatiles from its symbiont synergizes field captures of *X. glabratus* when presented concurrently with host-based attractants [66]. The ability to locate hosts that already support growth of appropriate fungal resources might be adaptive for *X. glabratus*. In addition to antennal chemoreception, females may detect cues with receptors located on other parts of their body. Since adult insects possess contact chemoreceptors on the base of the tarsae, females of *X. glabratus* may be able to ‘taste’ the host wood (detect non-volatile chemical constituents) as they walk across it. Tarsal receptors may also provide information regarding texture of the substrate.

In the present bioassays, females sometimes spent up to12 hours walking over the bolts before choosing a particular site to initiate boring; active boring most frequently occurred on the cut surface, apparently due to increased emissions of host volatiles, but potentially due to other physical or chemical properties. During the bioassays some females made shallow bore holes with their mandibles, apparently sampling the substrate at a particular site, before moving on to a new site. This behavior may result in detection of additional chemical cues by receptors (on maxillary and labial palpi) that surround the beetle mouthparts. These short-range cues may be positive or negative, signaling that the host is suitable (attractive) or not (repellent), and the interpretation of those cues may vary among individuals, as seen in the present bioassays (where results were expressed as the percentage of females boring).

Recently, it was reported that eucalyptol increased boring response of female *X. glabratus* in a paper arena bioassay, which may constitute the first known host-specific boring stimulant for a species of ambrosia beetle [39]. Evidence of a boring deterrent was seen with camphor tree in the present investigation; females were highly attracted to this species (due to high levels of α-copaene and α-cubebene), but a low percentage of females initiated boring after making physical contact. The species emits high levels of camphor, a monoterpene ketone suspected to be repellent to *X. glabratus* based on preliminary field trials [37]. In addition, GC-MS analysis detected a unique sesquiterpene (β-santalene, based on MS library match and previous detection in camphor tree [67]) that warrants further evaluation for repellency.

## Conclusions

Laurel wilt is firmly established in forests and agricultural ecosystems in the southeastern United States. The geographic range continues to expand naturally and through human transport, and movement to and along the U.S. Pacific Coast is possible, due to large populations of California bay laurel. Likewise, other species within the Lauraceae in Mexico, Central and South America, and the Caribbean Basin are at risk and may serve as conduits for the disease in areas where avocado is produced (as has occurred in Florida). To date, no cost-effective and efficacious measures have been identified to curtail the epidemic. It is apparent that a holistic approach is warranted for disease management, which will require a better understanding of the complex ecological and physiological interactions that occur among the insect vector(s), its fungal symbiont, and susceptible host trees. This report quantitated risk of attack by *X. glabratus* for the predominant U.S. species in the Lauraceae, and outlined a general scenario by which dispersing females locate and recognize appropriate host trees. It addition, semiochemicals were identified that are probable key components of the female's host discrimination process. This information should facilitate improvement of field lures for pest detection, and development of attract-and-kill bait stations for pest suppression.

## Supporting Information

Table S1 **Captures of female** ***Xyleborus glabratus*** **in field test 1.**
(XLSX)

Table S2 **Captures of female** ***Xyleborus glabratus*** **in field test 2.**
(XLSX)

Table S3 **Percentage of female** ***Xyleborus glabratus*** **boring into wood bolts in laboratory bioassay.**
(XLSX)

Table S4 **Terpenoid quantitation by gas chromatography-mass spectroscopy.**
(XLSX)

Table S5 **Dose-dependent electroantennogram responses of female** ***Xyleborus glabratus*** **.**
(XLSX)

Table S6 **Comparative electroantennogram responses of female** ***Xyleborus glabratus*** **.**
(XLSX)
